# Role of mTORC1 activity during early retinal development and lamination in human-induced pluripotent stem cell‐derived retinal organoids

**DOI:** 10.1038/s41420-022-00837-5

**Published:** 2022-02-08

**Authors:** Si Hyung Lee, Jung Woo Han, Jin Young Yang, Hyoung Oh Jun, Ji Hong Bang, Heejeong Shin, Ji Hye Choi, Jongwoo Lee, Sanjar Batirovich Madrakhimov, Kyung Hwun Chung, Hun Soo Chang, Jungmook Lyu, Tae Kwann Park

**Affiliations:** 1 Department of Ophthalmology, College of Medicine, Soonchunhyang University, Cheonan, Republic of Korea; 2 Department of Ophthalmology, Soonchunhyang University Hospital Bucheon, Bucheon, Republic of Korea; 3 Laboratory of Molecular Therapy for Retinal Degeneration, Hyangseol Medical Research Institute, Soonchunhyang University Hospital Bucheon, Bucheon, Republic of Korea; 4 Department of Interdisciplinary Program in Biomedical Science, Soonchunhyang University, Graduate School, Bucheon Hospital, Bucheon, Republic of Korea; 5 Department of Biological Sciences, Sungkyunkwan University, Suwon, Republic of Korea; 6 Akfa University Medical School, Tashkent, Uzbekistan; 7 Department of Anatomy and BK21 Four Project, College of Medicine, Soonchunhyang University, Cheonan, Republic of Korea; 8 Department of Medical Science, Konyang University, Daejun, Republic of Korea; 9 Ex Lumina Therapeutics and Technologies, Inc, Bucheon, Republic of Korea

**Keywords:** Induced pluripotent stem cells, Stem-cell research

## Abstract

Retinal organoids derived from human-induced pluripotent stem cells (hiPSC) are powerful tools for studying retinal development as they model spatial and temporal differentiation of retinal cell types. Vertebrate retinal development involves a delicate and coordinated process of retinal progenitor cell (RPC) differentiation, and the mammalian target of rapamycin complex 1 (mTORC1) has been reported to play a significant role in this complex process. Herein, using hiPSC-derived retinal organoids, we identify the time-dependent role of mTORC1 in retinal development, specifically in retinal ganglion cell (RGC) differentiation and the retinal lamination process, during the early stages of retinal organoid (RO) development. mTORC1 activity in ROs was the highest at 40 days of differentiation. MHY1485-induced hyperactivation of mTORC1 during this period resulted in a significant increase in the overall size of ROs compared to the untreated controls and rapamycin-treated Ros; there was also a marked increase in proliferative activity within the inner and outer layers of ROs. Moreover, the MHY1485-treated ROs showed a significant increase in the number of ectopic RGCs in the outer layers (indicating disruption of retinal laminar structure), with robust expression of HuC/D-binding proteins in the inner layers. These results demonstrate that mTORC1 plays a critical role in the development of hiPSC-derived ROs, especially during the early stages of differentiation.

## Introduction

The mature retina has a laminated structure consisting of various cell types, including photoreceptors, horizontal, bipolar, amacrine, and retinal ganglion cells (RGCs), as well as Muller glia [1, 2]. Development of this complex architecture requires both intra- and extra-cellular signaling [2]. Due to species-specific variations and differences between developmental stages, there is still much to be explored regarding the mechanisms of retinal development and lamination.

Mechanistic target of rapamycin (mTOR) is a conserved serine/threonine protein kinase that is responsible for cell proliferation, differentiation, survival, and autophagy regulation [3, 4], and several studies have shown its role in a number of pathological retinal conditions [5–9]. There are various sets of mTOR complexes; among them, mTORC1 is reported to be associated with the regulation of cell proliferation and growth, while mTORC2, although its functions are not well-known, is believed to be involved in cell shape change and motility [10]. Several previous studies have demonstrated the role of mTORC1 in retinal development in vivo. These studies support the theory that mTORC1 activation may facilitate the acceleration of retinal development, while its inhibition may lead to visual pathway dysfunction [11, 12]. However, other previously reported conflicting results suggest that mTORC1 activation may disrupt the proper development of visual pathways [13], leaving a question mark over the exact role of mTORC1 in retinal development.

Human-induced pluripotent stem cells (hiPSCs) are excellent research materials to study the development and differentiation of various cells [14–16], including those comprising the retina [17–21]. Recently, to overcome the limitations of two-dimensional in vitro experimentation, differentiation of hiPSCs into optic-cup-like three-dimensional retinal organoids (ROs) was successfully conducted [22–27], allowing the modeling of even the earliest stages of retinogenesis, such as retinal ganglion cell (RGC) development, and retinal lamination.

In this study, by modulating mTORC1 activity using rapamycin and MHY1485, we investigated the role of mTORC1 in the early stages of development of hiPSC-derived ROs. We mainly examined the effects on RGCs, which are known to be the earliest differentiated cell type within the ROs. Furthermore, we investigated the role of mTORC1 in retinal lamination during early retinogenesis, by observing displaced, ectopic RGCs in ROs.

## Results

### Development of RGCs in early ROs

**Fig. 1:**
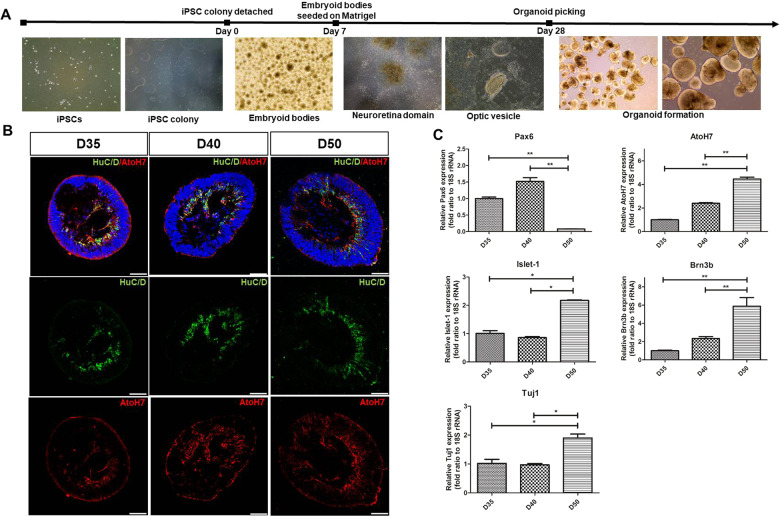
Formation of hiPSC-derived ROs and RGC development at an early stage. **A** Main steps of hiPSC-derived RO development in vitro: hiPSC colony, EB formation, neuroretinal domain, and ROs. **B** Immunofluorescence staining of HuC/D (green) and AtoH7 (red) of ROs at distinct differentiated time points. The expression of HuC/D and AtoH7 was gradually increased in the innermost layers of ROs from day 35 to day 60. **C** Quantitative RT-PCR results of PAX6, AtoH7, Islet-1, Brn3b, and Tuj1 levels of ROs at indicated time points. Data are expressed by mean ± S.E.M. *p < 0.05, **p < 0.01 by the Kruskal–Wallis test with post hoc analysis. Scale bar, 50 μm.

The formation of three-dimensional ROs was achieved by the culture of hiPSC-derived aggregates, which eventually formed embryoid bodies (EB) and then, fully grown ROs (Fig. 1A). After 28 days of differentiation, the ROs demonstrated a bright, stratified layer at their periphery, a defining morphological and phenotypic characteristic. The development of RGCs was assessed by the expression of various specific markers, as used in previous RO studies (HuC/D, AtoH7, Islet-1, and Brn3b) [27–30]. Using HuC/D and AtoH7 as representative RGC markers, we observed temporal and spatial patterns of RGC development in early ROs, from day 35 to day 50, with a time-dependent increase in both HuC/D and AtoH7 in the inner layers of ROs that are presumably made of RGCs (Fig. 1B). Quantitative real-time reverse transcription polymerase (RT-PCR) results showed that the expression levels of various markers of RGC, including AtoH7, Islet-1, Brn3b, and Tuj1 (class III beta-tubulin), were significantly increased in ROs at day 50 compared with those at day 35 (Fig. 1C). On the other hand, expression of Pax6, an early neural and retinal progenitor marker, showed an opposite trend in temporal pattern—its expression was high during the early periods of retinogenesis (day 35 and day 40) but decreased significantly at day 50 of differentiation.

### Temporal and spatial characteristics of mTORC1 activity in ROs

**Fig. 2:**
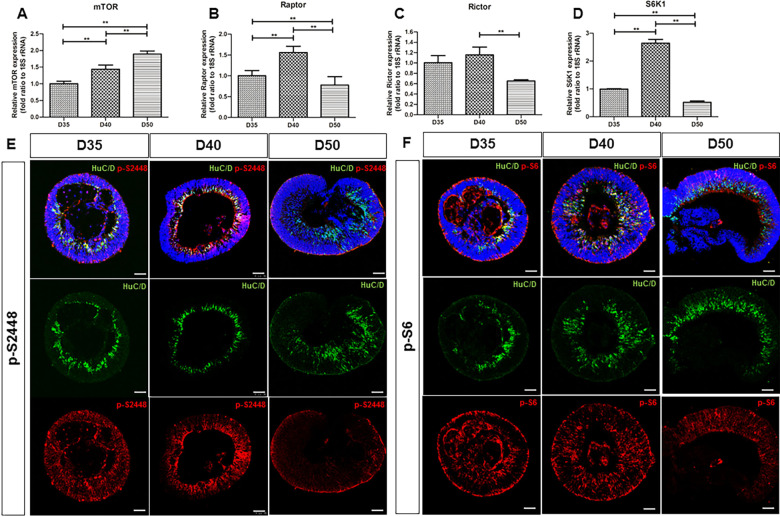
mTOR activity during early stages of RO development. Quantitative RT-PCR of **A** total mTOR, **B** Raptor, **C** Rictor, and **D** S6K1 using ROs at indicated days of development. Immunofluorescence staining of HuC/D (green) and p-S2448 (red) (**E**) or p-S6 (red) (**F**) of RO at indicated time points. After 35 days of differentiation, both p-S2448 and p-S6 expressions in ROs showed upregulation mainly in the inner layers which co-localized with HuC/D expression, while more robust expressions were found in the outer layers as well as the inner layers of ROs after 40 days of differentiation. Limited p-S2448 and p-S6 activities in outer layers of ROs with scarce co-localization with HuC/D^+^ cells were noted after 50 days of differentiation. **p < 0.01, by the Kruskal–Wallis test with *post hoc* analysis. Scale bar, 50 μm.

To investigate the dynamics of mTOR activity in ROs, we examined the relative expression of total mTOR, Raptor, and Rictor, which are subunits for mTORC1 and mTORC2, respectively, using quantitative RT-PCR. The results showed that total mTOR expression was significantly higher at day 50 than at day 35, with a pattern of gradual increase, according to experimental time points (*p* < 0.001, Fig. 2A). The Raptor expression level showed that mTORC1 activity was relatively high at day 40 (*p* < 0.001) and significantly lower at day 50 (*p* < 0.001, Fig. 2B). On the other hand, a non-significant increase in Rictor expression level was observed at day 40 compared to that at day 35 (*p* = 0.331, Fig. 2C). Given these results, we further confirmed the time-dependent activity of mTORC1 by evaluation of the expression level of S6 kinase (S6K1), which phosphorylates S6 (p-S6)—a downstream target of the mTORC1 pathway. Consistent with the expression pattern of Raptor, we found that S6 kinase expression was upregulated at day 40 (*p* < 0.001), and then significantly decreased at day 50 (*p* < 0.001, Fig. 2D). Furthermore, to evaluate the spatiotemporal pattern of mTORC1 activity in ROs, immunohistochemistry analyses using anti-p-S2448 and anti-p-S6 antibody were conducted. S2448 is predominantly phosphorylated domain for mTORC1 complex [31]. As with the temporal pattern of RT-PCR results, p-S2448 (Fig. 2E) and p-S6 expressions (Fig. 2F) were markedly upregulated at day 40 compared to that observed on days 35 and 50. p-S2448 and p-S6 expressions in the ROs, after 35 days of differentiation, were mainly distributed within the inner layers of ROs, presumably within the RGC layer, showing co-localization with HuC/D positive (HuC/D^+^) cells. At day 40, increased p-S2448 and p-S6 activities were observed in the entire RO layer. However, these expressions were limited in the outer layer, and p-S2448 and p-S6 immunofluorescence signal rarely co-localized with HuC/D^+^ cells in the ROs on day 50. These results suggest that mTORC1 signaling may be critical in regulating the proliferation and differentiation of cells, including RGCs, during the early stages of RO development.

### mTORC1 activity affects overall retinal development in early stages of RO differentiation

**Fig. 3:**
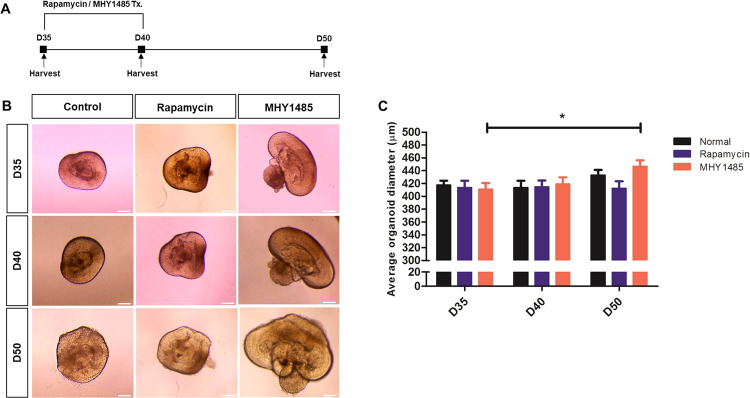
mTORC1 activity during early differentiation affects the overall size of ROs. **A** From day 35 to day 40, rapamycin and MHY1485, which are mTORC1 inhibitor and activator, respectively, were administered daily to investigate the role of mTORC1 in RO. **B** Light microscopic images of control, rapamycin-treated, and MHY1485-treated ROs at indicated time-points of development. **C** The average diameter of ROs at indicated time points. Data are expressed by mean ± S.E.M. *p < 0.05 by the Kruskal–Wallis test with post hoc analysis. Scale bar, 100 μm.

Given the results shown in Fig. 2 that mTORC1 is mainly activated during the early stages of RO differentiation, we manipulated mTORC1 activity using rapamycin (mTORC1 inhibitor) and MHY1485 (mTORC1 activator) during days 35 to 40 of differentiation to investigate its role in RO development (Fig. 3A). First, we analyzed the overall size of ROs. After 50 days of differentiation, the average RO diameter was smaller in the rapamycin treatment group than that in the control, without statistical significance (*p* = 0.890, Fig. 3B, C). On the other hand, ROs treated with MHY1485 were significantly larger compared to the control ROs (*p* = 0.031, suggesting that mTORC1 hyperactivation during the early period of differentiation may lead to hyperproliferation of ROs.

**Fig. 4:**
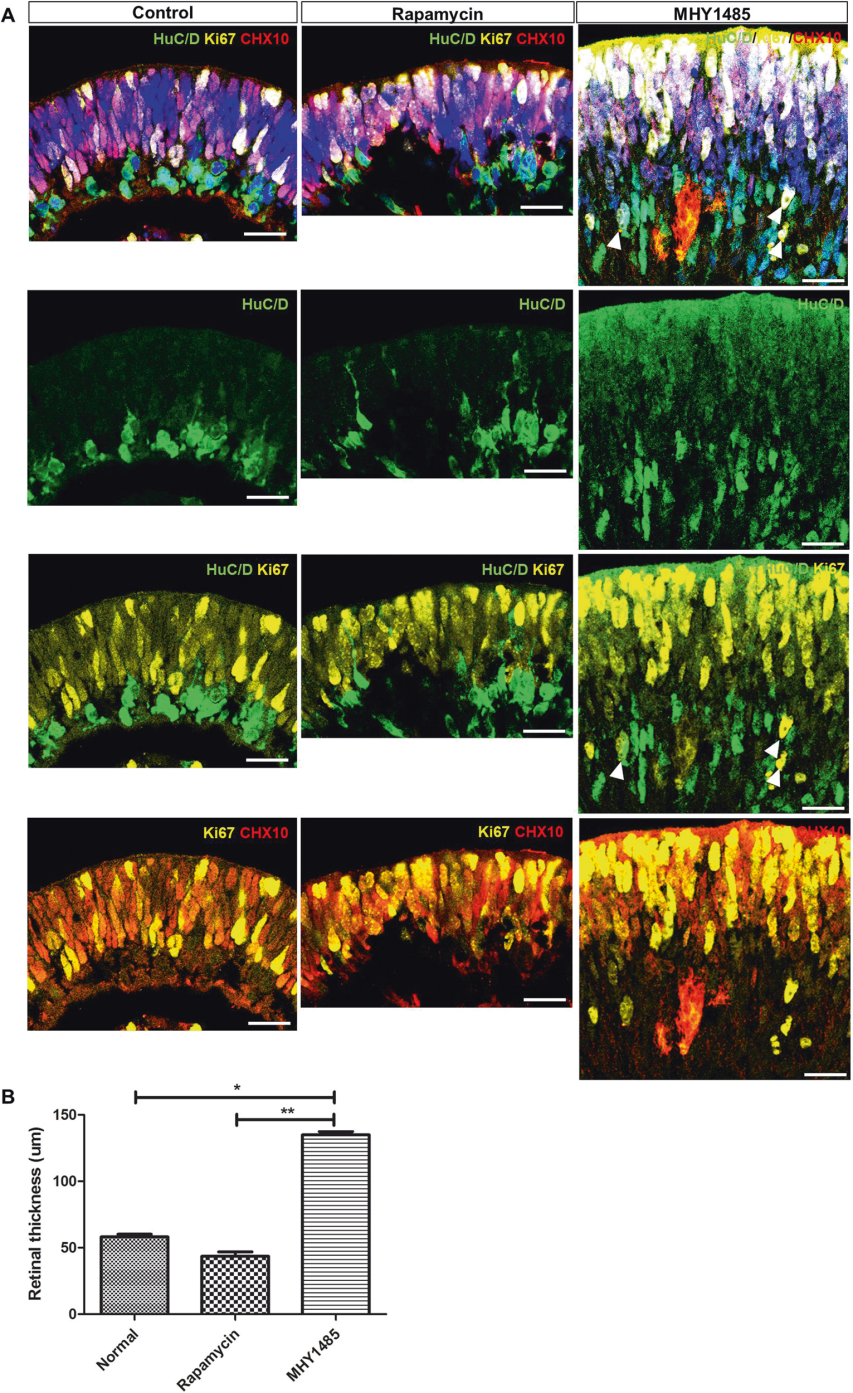
Temporal characteristics of proliferation and differentiation in ROs upon modulation of mTORC1 activity. **A** Comparison of proliferation and differentiation in control, rapamycin-treated, and MHY1485-treated ROs shown by immunostaining of Ki67 (yellow) and CHX10 (red) at 40 days of differentiation. In control and rapamycin-treated ROs, Ki67, and CHX10 expression was found in outer layers without any co-localization with HuC/D expression, while ROs with MHY1485 treatment showed upregulated expression of Ki67 and CHX10 throughout all RO layers, including RGC and outer neuroblastic cell layers. Disrupted retinal laminar structure was evident in MHY1485-treated ROs. **B** The average retinal thickness of in control, rapamycin-treated, and MHY1485-treated ROs. Arrowheads indicate co-localization of HuC/D and Ki67 expression. Data are expressed by mean ± S.E.M. *p < 0.05, **p < 0.01 by the Kruskal–Wallis test with *post hoc* analysis. Scale bar, 25 μm.

We also investigated differences in the temporal patterns of cell development among control, rapamycin-treated, and MHY1485-treated ROs by immunostaining for Ki67 and CHX10 (well-known proliferation and retinal progenitor markers). After 40 days of differentiation, expression of Ki67 and CHX10 was detected in the outer layers of untreated control ROs, which were considered to be neuroblastic cell layers, whereas these markers were barely detectable in the RGC layer—identified by HuC/D expression (Fig. 4A). Spatial patterns of Ki67 and CHX10 expression were also similar in rapamycin-treated ROs, showing reduced outer layer thickness compared to that observed in control ROs. However, ROs treated with MHY1485 showed upregulated expression of Ki67 throughout all RO layers, including the RGC and outer neuroblastic cell layers. Moreover, MHY1485-treated ROs displayed disrupted retinal laminar structures with significantly increased retinal-layer thickness (*p* < 0.001, Fig. 4B). These findings indicate that mTORC1 hyperactivation may result in robust cell proliferation throughout all the RO layers, including proliferation of the already differentiated RGCs.

### Early RGC development and retinal lamination are sensitive to mTORC1 activity

**Fig. 5:**
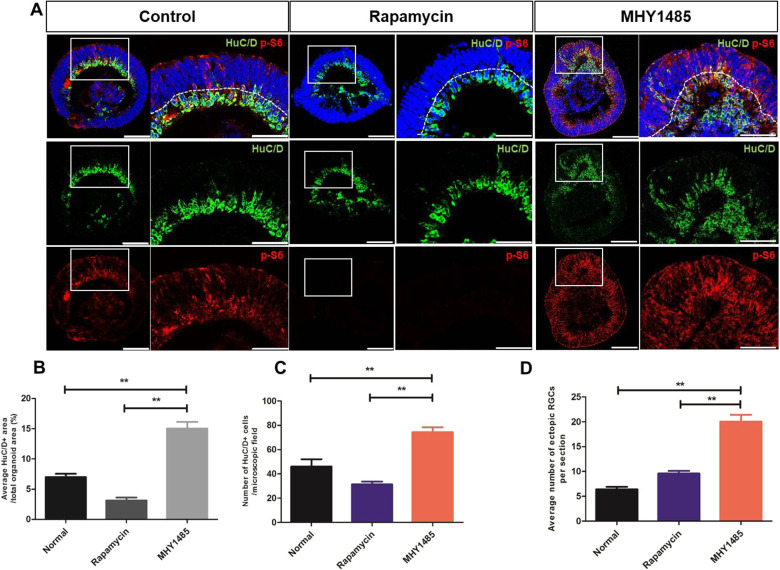
RGC development and retinal lamination within ROs at 40 days of differentiation after rapamycin or MHY1485 treatment. **A** RGC development in control, rapamycin-treated, and MHY1485-treated ROs shown by immunostaining of HuC/D (green) and p-S6 (red) at 40 days of differentiation. **B**–**D** Quantification of the ratio of average HuC/D^+^ area, number of HuC/D positive cells, and number of ectopic RGCs in control, rapamycin-treated, and MHY1485-treated ROs. Data are expressed by mean ± S.E.M. **p < 0.01, by the Kruskal–Wallis test with post hoc analysis. Scale bar in RO whole-image: 100 μm, scale bar in RO magnified image: 50 μm.

RGCs are the first cell type to be differentiated within the innermost layers of the retina. We therefore investigated whether mTORC1 activity may affect RGC development in ROs. Within 40 days of differentiation, HuC/D^+^ cells, presumably RGCs, were identified in the innermost layers of control ROs (Fig. 5A). Upon rapamycin treatment, fewer HuC/D^+^ cells were detected within the inner layers of ROs, with barely detectable p-S6 activity. On the other hand, MHY1485-treated ROs demonstrated robust expression of HuC/D in the innermost layers during the same experimental period, suggesting increased differentiation of what are presumably RGCs. Evaluation of the average number of HuC/D^+^ cells in a given area of ROs revealed that the HuC/D^+^ cell population was significantly increased in MHY1485-treated ROs than in control and rapamycin-treated ROs (*p* < 0.001, Fig. 5B, C). To further investigate the effect of mTORC1 activity on retinal lamination, we counted the number of displaced HuC/D^+^ cells detected in the outer layers of ROs. Compared to that in the control and rapamycin-treated ROs, the average number of ectopic RGCs was significantly higher in MHY1485-treated ROs (*p* < 0.001, Fig. 5D), while the difference between the control and rapamycin-treated ROs did not show statistical significance. These results indicate that mTORC1 activation may play an important role in retinal lamination, one of the critical processes in retinogenesis.

**Fig. 6:**
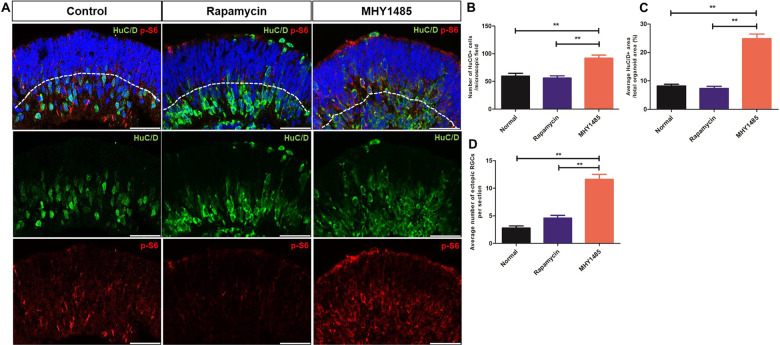
RGC development and retinal lamination in ROs at 50 days of differentiation after rapamycin or MHY1485 treatment. **A** RGC development in control, rapamycin-treated, and MHY1485-treated ROs shown by immunostaining of HuC/D (green) and p-S6 (red) at 50 days of differentiation. **B**–**D** Quantification of the ratio of average HuC/D area, number of HuC/D positive cells, and number of displaced RGCs in control, rapamycin-treated, and MHY1485-treated ROs. Data are expressed by mean ± S.E.M. **p < 0.01, by the Kruskal–Wallis test with post hoc analysis. Scale bar: 50 μm.

Comparing HuC/D^+^ cells among experimental groups after 50 days of differentiation demonstrated similar results, showing that the presumable RGCs were the most abundant in the inner layers of MHY1485-treated ROs than in the inner layers of control and rapamycin-treated ROs (Fig. 6A, B). The average number of ectopic RGCs was also significantly higher in MHY1485-treated ROs (*p* < 0.001), as observed in ROs from 40 days of differentiation.

**Fig. 7:**
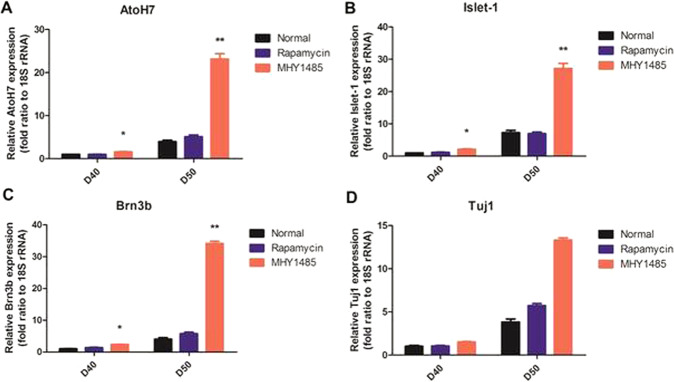
Quantitative RT-PCR analysis of ROs for RGC markers upon rapamycin and MHY1485 treatment. Relative AtoH7 (**A**), Islet-1 (**B**), Brn3b (**C**), and Tuj1 (**D**) expression level in ROs treated with rapamycin or MHY1485 and untreated controls. MHY1485-treated ROs showed significantly increased expression of RGC markers Data are expressed by mean ± S.E.M. **p < 0.01, by the Kruskal–Wallis test with post hoc analysis.

Quantitative RT-PCR analysis showed significantly increased expression of various RGC markers, including AtoH7, Islet-1, Brn3b, and Tuj1, in MHY1485-treated ROs compared to the control ROs after 40 and 50 days of differentiation (Fig. 7A–D). Moreover, representative AtoH7 immunostaining showed similar pattern demonstrating upregulation of AtoH7 in MHY1485-treated ROs, mainly in inner layers (Supplementary Fig. 1). These observations further support the findings from HuC/D immunostaining.

## Discussion

Over the recent decades, considerable progress has been made to improve the understanding of retinal development. It is widely known that many developmental signaling pathways are involved in retinal development. The mTOR pathway is a ubiquitous nutrient-sensing intracellular pathway that promotes the growth and survival of cells, mainly by regulating protein synthesis. mTORC1 regulates neurogenesis in the eye, and precise spatiotemporal regulation of its activity is central to normal development of the retina [11, 32, 33], including RGC development [12, 13, 34]. However, to the best of our knowledge, there have been no reports investigating the role of mTORC1 pathways in retinal development using hiPSC-derived ROs. Herein, we used hiPSC-derived ROs to demonstrate the potential role of mTORC1 in human retinal development, especially the development of RGCs (the first cell type to be specified in the innermost layers of the retina), as well as in retinal lamination. Within ROs, mTORC1 activity was mainly detected during the early stages of differentiation—at 40 days. By modulating mTORC1 activity with rapamycin or MHY1485 during this period, we found that hyperactivation of mTORC1 resulted in increased proliferative activity, showing increased overall size of ROs and thickness of retinal layers. Moreover, mTORC1 hyperactivation in ROs induced robust differentiation of neuronal cells presumed to be RGCs in the inner RO layers, which was confirmed by various RGC markers, immunohistochemistry (IHC), and quantitative RT-PCR analysis. Moreover, mTORC1 hyperactivation resulted in the disruption of lamination of retinal layers compared to rapamycin-treated and untreated control ROs.

In this study, we observed a temporal pattern of mTORC1 expression in ROs during retinal development, mainly activated during 35 to 40 days of differentiation. This observation coincided with the temporal activity of Pax6 (a representative indicator for RPCs), which also inversely coincided with the activity of various RGC markers, gradually increasing until 60 days of differentiation, as reported in previous studies [27, 35]. One previous in vivo study showed that mTORC1 activity was upregulated in the differentiating ganglion cells of a developing chick retina, while it was relatively low in mature, normal retina [36]. Such findings indicate that mTORC1 activity during the early period of RO development may play a significant role in determining neural cell fate. Furthermore, we observed that p-S6 activity was high, mainly in the innermost layers of ROs on 35 and 40 days of differentiation, especially in HuC/D^+^ cells, suggesting that mTORC1 was involved in the initiation and differentiation of RPCs along the RGC lineage during the early stages of RO development.

Indeed, we found that hyperactivation of mTORC1 during the early stages of RO development resulted in an increased number of HuC/D^+^ cells in the inner layers of ROs, which were considered to be RGCs, compared to the untreated control and rapamycin-treated ROs. These observations suggest that mTORC1 may play an important role in controlling decisions regarding cell fates and cell specification during the early period of RPC differentiation, which corroborates with previous findings from both *Drosophila* and mouse studies [11, 13, 32, 37]. These studies reported that precocious activation of mTORC1 led to hyperproliferation of neuronal cells ahead of their regular developmental schedule. Considering that early hiPSC-derived RO differentiation encompasses a period of RPC proliferation and production of RGC within the innermost layer [27, 38], our results from ROs support previous in vivo findings that appropriately timed expression of mTORC1 is required for normal control of the fates of RPCs, and that any disturbances in this process may lead to anomalous retinal development.

Previously, Choi et al. [11] demonstrated that Raptor-ablation in the RPCs of mouse retina resulted in the failure of cell cycle progression during retinal development, which eventually led to decreased retinal thickness. A more recent study using Lhx2-cre-mediated Raptor deletion mouse model showed similar results, demonstrating a reduction in the total retina size and thickness [12]. Moreover, a study from the same group reported that TSC1-ablation in mice during early embryonic development showed the enlarged overall size of eyes compared to control littermates, indicating that mTORC1 signaling controls overall organ size in the nervous system [13]. In this study, using hiPSC-derived ROs, we observed consistent findings showing that the inhibition of mTORC1 during the early phase of RO development resulted in an overall decrease in the size and thickness of retina, and vice versa when treated with MHY1485. Our findings therefore model previous findings from in vivo studies, that mTORC1 regulates cell growth and proliferation in the retina.

Interestingly, in this study, mTORC1 hyperactivation during the early phase of differentiation resulted in the increased number of both Ki67-labeled proliferating cells and CHX10-positive RPCs. However, these findings were different from a recent publication reporting that mTORC1 hyperactivation in mouse retina resulted in increased proliferating cell population, but not in the total number of RPCs [11]. Such discrepancies may be due to the innate properties of ROs characterized by relatively persistent CHX10 expression until 60 days of differentiation [27], as well as different experimental designs (in vitro vs. in vivo).

Retinal lamination is an essential developmental step for unique retinal stratification, and alteration of this process may lead to impaired visual function [39]. In this study, we observed that the dysregulation of mTORC1 activity, especially hyperactivation of mTORC1, caused an increase in the number of ectopic RGCs in the outer layers of RO at 40 and 50 days of differentiation, meaning that mTORC1 hyperactivation may result in disruption of retinal laminar organization. Our results are in agreement with previous reports that mTORC1 hyperactivation may lead to significant disruption of retinal lamination [11, 13]. On the other hand, rapamycin-treated ROs did not show obvious disruption of the lamination process, and results did not differ significantly from those of untreated control ROs. Considering that in normal vertebrate retina, neuronal migration occurs after neuronal cell generation [40], we may infer from our results that a decrease in mTORC1 activity may be a necessary step for proper retinal lamination. However, it should be noted that in the present study, mTORC1 inhibition was performed at the time when RGCs were already in existence within the inner RO layers, while previous studies used knock-out in vivo models mimicking mTORC1 inhibition from the time of RPC differentiation. Furthermore, we did not identify changes in retinal lamination during later stages of the current study, and therefore further in-depth studies are needed.

In summary, our study revealed that mTORC1 activity during early retinal differentiation is closely correlated with the process of human retinal development, especially for RGC differentiation and retinal lamination, as demonstrated with hiPSC-derived ROs. Our findings allowed insight into previously unexplored early-phase development of human retinas, further establishing a basis for the role of mTORC1 in human retinal neuronal cell development. Moreover, our results also outline the feasibility of using hiPSC-derived ROs as a tool for translational research on retinal development.

## Materials and methods

### hiPSC cultures

American Type Culture Collection (ATCC)-DYR0100 hiPSCs (ACS-1011, ATCC, Manassas, VA) were used in our experiment. The hiPSCs were maintained on vitronectin-coated dishes (Gibco CTS vitronectin; Thermo Fisher Scientific, Waltham, MA) in Essential 8 medium (Thermo Fisher Scientific). Cells were routinely cultured at 37 °C in a standard 5% CO_2_/95% air incubator, with a daily medium change. Upon reaching ~70–80% confluency, cells were mechanically passaged with EDTA every 5–7 days. Detached cell aggregates were collected in Essential 8 (E8) medium and carefully pipetted up and down to obtain uniform suspension of cell aggregates; these were re-plated at a ratio of 1/10 to 1/60, depending on the confluence.

### Differentiation into three-dimensional ROs

The differentiation of ROs was performed as described previously [38]. hiPSCs were maintained in 6-well vitronectin plates (Thermo Fisher Scientific). E8 media (Thermo Fisher Scientific) were all successfully used to maintain the health and pluripotency of the hiPSCs. hiPSCs were detached by treatment with human PSC passaging reagent ReLeSR (STEMCELL Technologies, Vancouver, Canada), dissociated into single cells and cultured in a low attachment dish with ROCK inhibitor Y27632 and a 3:1 mixture of E8 media and neural induction medium (NIM) containing DMEM/F12 (1:1, Thermo Fisher Scientific), 1% N-2 supplement, 0.1 mM non-essential amino acids (NEAAs), and 2 µg/ml heparin (Thermo Fisher Scientific) at day 0 to induce EB formation. EBs were gradually transitioned into NIM by replacing the E8 medium with NIM. On day 7, EBs were seeded to a 6-well plate containing NIM with 10% fetal bovine serum (FBS) at an approximate density of 30–40 EBs/well. On day 16, the medium was changed from NIM to retinal differentiation medium (RDM) containing DMEM/F12 (3:1, Welgene), 2% B-27 supplement without vitamin A (Thermo Fisher Scientific), 0.1 mM NEAAs, and penicillin/streptomycin (Thermo Fisher Scientific). Around day 18–25, the loosely adherent central portions of the neural clusters were lifted using a P1000 pipettor. The collected aggregates were cultured in suspension with RDM, which allowed them to form three-dimensional optic-cup structures. For long-term suspension culture, the medium was supplemented with 10% FBS (Thermo Fisher Scientific), 100 mM Taurine (Sigma-Aldrich, St. Louis, MO), and 2 mM GlutaMAX (Thermo Fisher Scientific), beginning on day 35. The cell medium was changed every 2–3 days until the desired stage was reached.

### Rapamycin and MHY1485 treatment

Rapamycin and MHY1485 were prepared at final concentrations of 100 nM and 10 μM, respectively. From 35 days of differentiation, organoids were transferred to the wells of a 96-well plate and treated with rapamycin and MHY1485 until 40 days of differentiation. The medium was subsequently changed with fresh RDM with rapamycin and MHY1485 every day during the treatment period.

### Immunohistochemistry

The ROs were fixed in 4% paraformaldehyde at room temperature for 30 min and cryoprotected in 15% sucrose for at least 2 h, followed by 30% sucrose overnight, before being embedded in optimum cutting temperature (OCT) compound. The OCT blocks were sectioned at 10 μm thickness and incubated at room temperature for at least 1 h, before immunostaining or storage at −80 °C. Slides were rinsed in 0.1% Triton X-100 (Sigma-Aldrich) in PBS and blocked with 5% donkey serum in PBS for 2 h, and incubated overnight at 4 °C with primary antibodies diluted in blocking solution. Primary antibodies were used at the following dilutions: HuC/D (1:300; A-21271, Thermo Fisher Scientific), Ki-67 (1:300; ab16667, Abcam, Cambridge, UK), CHX10 (1:100; X1179P, Exalpha Biologicals, Shirley, MA), p-S2448 (1:250; 44-1125G, Invitrogen, Waltham, MA), p-S6 ribosomal protein (1:500; 5346, Cell Signaling Technology, Danvers, MA), AtoH7 (1:500; PA5-35859, Thermo Fisher Scientific). Species-specific secondary antibodies, conjugated with Alexa Fluor 488 or 568, were diluted in antibody dilution buffer and incubated at room temperature for 2 h. Hoechst® 33342 (H1399, Invitrogen) was used for nuclear staining. Fluorescence images were captured using a confocal microscope (DMI8; Leica Camera, Wetzlar, Germany).

### Quantitative reverse transcription-PCR

The mRNA levels of mTOR, AtoH7, Islet-1, Brn3b, and Tuj1-2 were measured by quantitative RT-PCR. Total mRNA was prepared from 3 to 5 ROs using TRIzol reagent (Invitrogen). mRNA (2 μg) was reverse transcribed using Superscript III (Invitrogen). Quantitative RT-PCR analysis was conducted using an SYBR Green kit (Invitrogen), and the samples were quantified by amplifying GAPDH as an internal control for each sample. The PCR amplification was performed in triplicate using specific primer pairs, as listed in Table 1.

**Table 1:** List of primers used for quantitative RT-PCR.

Gene	Primer sequence	
	Forward	Reverse
Pax6	5′-CGG AGT GAA TCA GCT CGG TG-3	5′-CCG CTT ATA CTG GGC TAT TTT GC-3
AtoH7	5′-GTG GGG CCA GGA TAA AAA GC-3′	5′-CGG GTC AGA GCC ATG TAG4-3′
Islet-1	5′-GCG GAG TGT AAT CAG TAT TTG GA-3′	5′-GCA TTT GAT CCC GTA CAA CCT-3′
Brn3b	5′-TGA CAC ATG AGC GCT CTC ACT TAC-3′	5′-ACC AAG TGG CAA ATG CAC CTA-3′
Tuj1	5′-GGC CAA GGG TCA CTA CAC G-3′	5′-GCA GTC GCA GTT TTC ACA CTC-3′
Rictor	5′-GGAAGCCTGTTGATGGTGAT-3′	5′-GGCAGCCTGTTTTATGGTGT-3′
Raptor	5′-ACTGATGGAGTCCGAAATGC-3′	5′-TCATCCGATCCTTCATCCTC-3′
mTOR	5′-ACA GAG TTG GAG CAC AGT GG-3′	5′-GGC CAC TAA CCT GTG CCA AT-3′
S6K1	5′-GCACAGCAAATCCTCAGACA-3′	5′-GATGCTTCCCCACTCATTGT-3

### Image analyses and statistical analyses

The brightfield color images of retinal organoids were taken with an EVOS XL Core Cell Imaging System (Thermo Fisher Scientific). Quantification of the size and the HuC/D^+^ cell number of ROs were conducted using Image-J software, and the means of quantified results with standard deviations were obtained using GraphPad Prism 5 software (GraphPad Software, San Diego, CA). The non-parametric Kruskal–Wallis test with post hoc analyses was used to determine statistically significant differences among different time points and treatment groups. Multiple biological replicates were used for the analysis at each time point for each group (*n* = 5). Sample size and power calculation were performed using Gpower v.3.1 (Statistical analyses were conducted using SPSS for Windows software (ver. 20.0; SPSS Inc., Chicago, IL). Differences at *p* < 0.05 were considered statistically significant.

## Supplementary information

Supplementary Figure 1

## Data Availability

The datasets generated and analyzed during the current study are available from the corresponding author on reasonable request.
