# Supplementary material for: Role of mTORC1 activity during early retinal development and lamination in human-induced pluripotent stem cell‐derived retinal organoids
Source: Cell Death Discov. 2022 Feb 8;8:56. doi: 10.1038/s41420-022-00837-5 (PMC8826382; doi:10.1038/s41420-022-00837-5)
Supplement: Supplementary file 1 — Supplementary Figure 1 [file 41420_2022_837_MOESM1_ESM.docx]

**
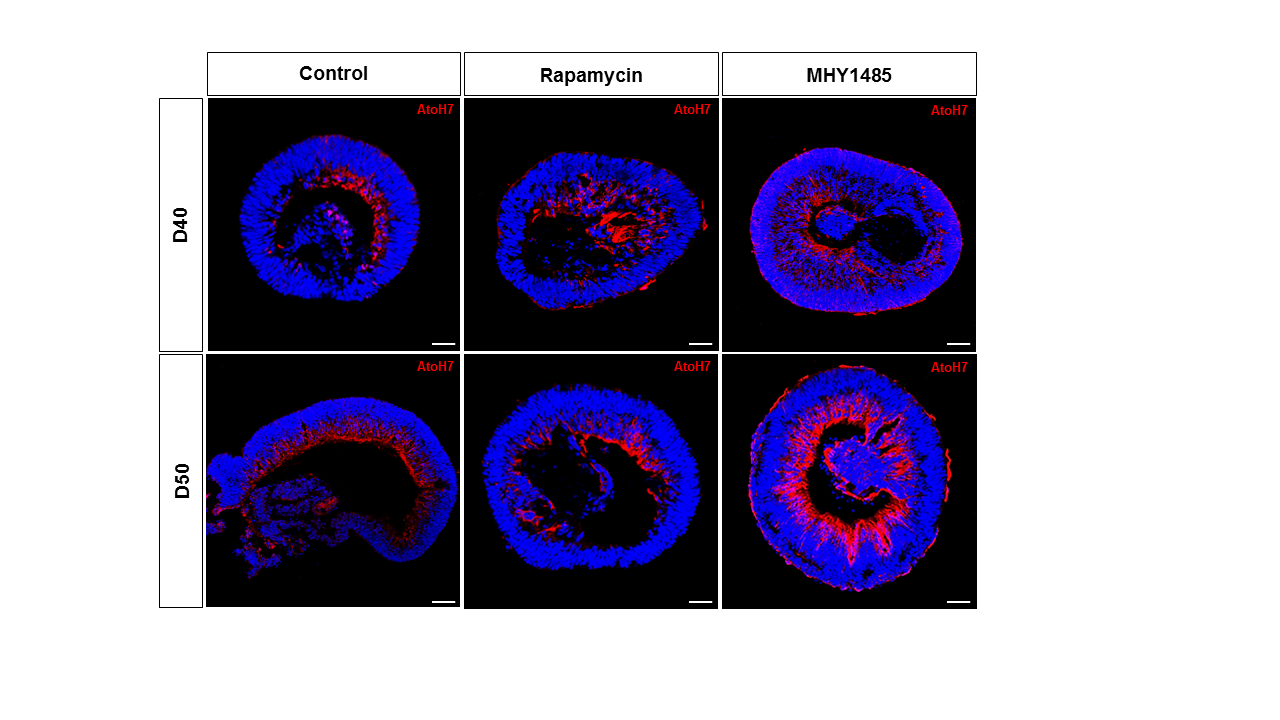
**

**Supplementary Fig. 1. Representative images of AtoH7 immunostaining from control, rapamycin-treated, and MHY1485 treated ROs.**

Compared to control and rapamycin-treated ROs, MHY1485-treated ROs showed upregulation of AtoH7 expression, mainly in inner layers, at both 40 days and 50 days of differentiation. Scale bar, 50 μm.
